# Molecular Mechanisms and Regulatory Pathways Underlying Drought Stress Response in Rice

**DOI:** 10.3390/ijms25021185

**Published:** 2024-01-18

**Authors:** Anjing Geng, Wenli Lian, Yihan Wang, Minghao Liu, Yue Zhang, Xu Wang, Guang Chen

**Affiliations:** 1 Institute of Quality Standard and Monitoring Technology for Agro-Products of Guangdong Academy of Agricultural Sciences, Guangzhou 510640, China; 2 Key Laboratory of Testing and Evaluation for Agro-Product Safety and Quality, Ministry of Agriculture and Rural Affairs, Guangzhou 510640, China; 3 Guangdong Provincial Key Laboratory of Quality & Safety Risk Assessment for Agro-Products, Guangzhou 510640, China

**Keywords:** rice, drought, regulatory pathways, molecular mechanisms

## Abstract

Rice is a staple food for 350 million people globally. Its yield thus affects global food security. Drought is a serious environmental factor affecting rice growth. Alleviating the inhibition of drought stress is thus an urgent challenge that should be solved to enhance rice growth and yield. This review details the effects of drought on rice morphology, physiology, biochemistry, and the genes associated with drought stress response, their biological functions, and molecular regulatory pathways. The review further highlights the main future research directions to collectively provide theoretical support and reference for improving drought stress adaptation mechanisms and breeding new drought-resistant rice varieties.

## 1. Introduction

Crop growth and yield are inhibited by a complex growth environment caused by climate change [1]. Drought is one of the most harmful abiotic stresses that hinder agricultural productivity globally, resulting in a yield decrease of up to 50–70% [1]. The World Health Organization (WHO) estimates that drought affects the livelihoods of about 55 million people globally every year [2]. The WHO further projects that about 700 million people will be at risk of displacement due to drought by 2030 [2].

Rice is a staple food for 350 million people globally. The global population is expected to rise to 10 billion by 2050, and it will require 852 million tons of rice by 2035 [3,4]. Notably, 90% of the world’s rice is cultivated in Asia [5]. The water requirement for rice is two-to-three times higher than that of dryland grains because of its growth characteristics [6]. For instance, the production of 1 kg of rice requires 1432 L of irrigation water [7]. Water is thus a major factor that affects rice yield. In Asia, 5 million hectares of upland rice and 34 million hectares of lowland rice are exposed to drought stress, leading to reduced yield, which poses a great threat to global food security [8].

Drought stress affects the expression of numerous genes, causing changes in the morphology, physiology, biochemistry, and other aspects of rice, which inhibit its growth, development, and yield. Currently, there is no feasible method for improving rice yield under drought conditions. Cultivating drought-resistant rice is an effective strategy to meet the growing food needs of developing and underdeveloped countries. A comprehensive understanding of the physiological and molecular mechanisms of rice response to drought stress lays a theoretical foundation for the creation of drought-resistant rice [9]. This review expounds on the research progress of different organs of rice in response to drought stress at the morphological, physiological, and molecular levels. The review details the drought-related genes in rice, their biological functions in drought response, and the molecular regulatory pathways involved during drought response as a basis for stabilizing the yield of rice during drought stress.

## 2. Morphological, Physiological, and Biochemical Changes in Rice in Response to Drought Stress

Drought inhibits cell elongation and expansion [10], which affects root growth, thus reducing nutrient absorption that leads to growth retardation, decreased leaf water potential and net photosynthesis, and spikelet sterility [11]. Drought also induces the production of reactive oxygen species and enhances the oxidation of lipids and proteins, which affects the biosynthesis of osmotic regulators (proline, betaine, sorbitol, and mannitol, among other regulators) and destroys the redox homeostasis and ion balance [12,13]. Moreover, it affects the signal perception and transduction mediated by MAPKs and Ca^2+^, the expression of drought stress response genes and ABA synthesis genes, and the synthesis of aquaporins [14], which significantly affect the growth and development of rice, resulting in a decrease in biomass and yield. Table 1 outlines the morphological, physiological, and biochemical changes in different organs of rice during drought stress.

## 3. Genes Associated with Drought Stress Response and Their Biological Functions in Rice

**Figure 1:**
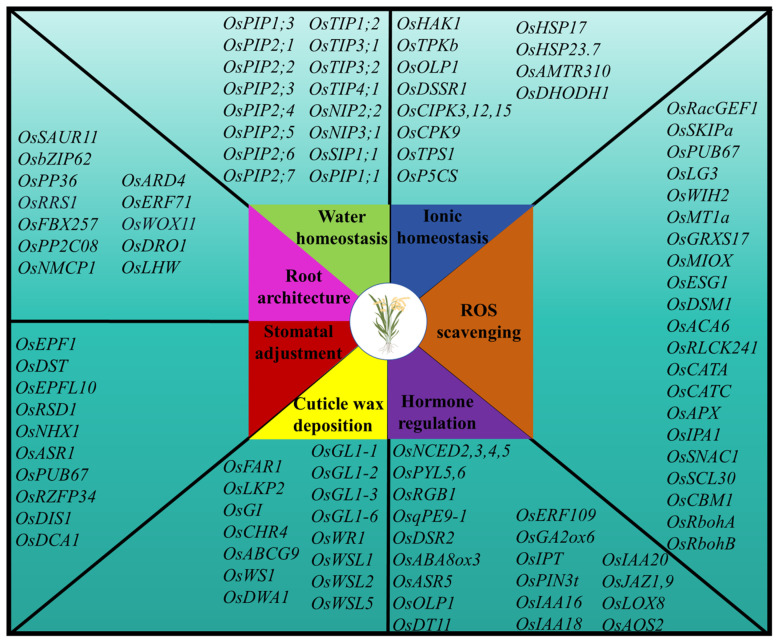
Functional genes that regulate drought tolerance in rice by regulating water and ROS homeostasis, osmotic substances and hormone content, cuticular wax deposition, stomatal density or opening and closing, and root architecture.

The National Rice Data Center (https://www.ricedata.cn/ accessed on 2 April 2020) postulates that 262 functional genes related to rice drought resistance were successfully cloned by 2020. The genes are distributed on 12 chromosomes [31], and their main functions are maintaining water and ROS homeostasis, osmotic regulation, regulating hormone content, cuticular wax deposition, stomatal density or opening and closing, and improving root architecture [32,33] (Figure 1).

### 3.1. Maintenance of Water Homeostasis

Aquaporins (AQPs) play a role in the transmembrane transport of water to maintain water homeostasis. The transcellular movement of water is strictly controlled by the number and activity of AQPs in the membrane. Notably, it is associated with the transport, gating, and degradation of AQPs [34]. In rice, there are 33 AQP isoforms composed of 11 plasma membrane intrinsic proteins (PIPs), which are the most abundant AQPs on the plasma membrane, 10 vacuolar intrinsic proteins (TIPs), 10 nodulin 26-like intrinsic proteins (NIPs), and 2 small intrinsic proteins (SIPs) [35]. Some of these AQPs respond to drought stress [36]. The regulation of AQPs in rice potentially plays a role in drought resistance because AQPs are closely associated with plant water homeostasis [37]. Nguyen et al. (2013) studied the response of root AQPs to drought conditions and found that *OsTIP1;2*, *OsTIP3;1*, *OsTIP3;2*, and *OsTIP4;1* were up-regulated, while *OsNIP2;2*, *OsNIP3;1*, and *OsSIP1;1* were down-regulated [38]. PEG6000 simulates drought stress, inducing the expression of *OsPIP1;1*, *OsPIP2;5* and *OsPIP2;7* in roots and *OsPIP2;3* in leaves. However, it inhibits the expression of *OsPIP2;1*, *OsPIP2;5*, and *OsPIP2;6* in leaves [39]. The overexpression of *OsPIP1;1* or *OsPIP2;2* in *Arabidopsis* improves its drought tolerance [40]. The overexpression of *OsPIP2;2* promotes H_2_O transport, thus effectively protecting rice cells from increased electrolyte leakage, proline, and polyamine concentrations caused by physiological drought stress, and thereby enhancing the drought tolerance of transgenic rice [41]. *OsPIP1;3* enhances drought tolerance by regulating water movement on the plasma membrane during a water deficit. For instance, *SWPA2:OsPIP1;3*-overexpressing transgenic rice has a better water status under water deficit conditions [36]. Liu et al. (2020) identified an *OsPIP1;3* allele from drought-resistant rice varieties that is mainly expressed in rice roots and strongly responds to drought stress. An ectopic expression of *OsPIP1;3* in tobacco promotes vegetative growth and water absorption, thus improving the drought resistance of transgenic tobacco [42]. The overexpression of *OsPIP2;1* increases the cell membrane permeability of yeast, root hydraulic conductivity, total root length, root surface area, root volume, and root tip number of rice. However, these parameters were significantly reduced with the inhibition of *OsPIP2;1* expression, thus confirming its sensitivity to drought stress [43]. Nada et al. (2020) overexpressed *OsPIP2;4* in the *japonica* rice variety Giza178 and *indica* rice variety IR64, but observed different responses to drought stress because of the differences in the root traits and aquaporin regulation between the two transgenic varieties. This finding strongly suggests that the influence of plant internal factors should be comprehensively considered when optimizing AQP gene expression patterns [44].

### 3.2. Osmotic Regulation

During drought, rice maintains the balance of osmotic pressure by increasing the concentration of intracellular osmotic substances, thereby slowing down or hindering cell dehydration, which maintains a normal physiological metabolism of cells to resist drought stress. Osmotic substances are divided into two categories: inorganic ions entering the cell from the outside and organic solutes synthesized in the cell. The organic solutes include soluble sugars (e.g., sucrose and trehalose), alcohols (e.g., mannitol and sorbitol), free amino acids (e.g., proline), and betaine [45].

K^+^ participates in osmotic regulation and maintains cell turgor, which is closely associated with water balance and water use efficiency [46,47,48]. Optimizing K^+^ absorption is thus an important response of plants to drought stress [49,50]. Drought inhibits K^+^ absorption by inhibiting root growth, resulting in a decrease in K^+^ accumulation and further reducing the drought tolerance of rice [51]. *OsHAK1* is a drought-responsive gene that regulates K^+^ homeostasis to improve drought resistance in rice [52]. The expression of *RAA1* driven by the *OsHAK1* promoter promotes root growth, potassium accumulation, and tolerance to water stress in rice [51]. A constitutive overexpression of the vacuolar K^+^ channel gene *OsTPKb* promotes the growth of rice under K^+^-deficient conditions and improves the tolerance of rice to osmotic and drought stresses [53].

Transferring some vital osmotic regulation substance synthesis genes into rice via transgenic methods can significantly improves the drought resistance of rice [54,55]. The overexpression of *OsOLP1*, *OsDSSR1*, *OsCIPK03*, *OsCIPK12*, *OsCIPK15*, *OsTPS1*, *OsCPK9*, *OsP5CS*, *OsHsp17.0*, and *OsHsp23.7* promotes the accumulation of osmotic substances such as proline, soluble sugar, and trehalose, thereby significantly improving the drought tolerance of rice [56,57,58,59,60,61,62]. Of note, the free proline content of *OsAMTR310* transgenic plants is 1.5–2 times that of the wild type (WT) during drought, which enhances the drought tolerance of rice [63]. Δ1-pyrroline-5-carboxylate synthetase (P5CS) cDNA from *Vicia faba* causes an excessive production of P5CS enzyme and accumulation of proline when introduced into the rice genome, thereby increasing the biomass of transgenic rice plants during drought stress [61]. An overexpression of *OsDHODH1* increases uridine 5′-monophosphate and proline content promotes the synthesis of osmotic substances and proteins, and reduces the damage of rice cells caused by drought stress [64]. The transfer of the *adc* gene from *Datura stramonium* into rice leads to an increase in the accumulation of putrescine and promotes the synthesis of spermidine and spermine during drought stress, thus enhancing drought resistance [65].

### 3.3. Maintenance of ROS Homeostasis

During drought stress, organelles such as chloroplasts and mitochondria, which undergo electron transfer, produce excessive reactive oxygen species (ROS), causing damage to the membrane lipid structure and cell function. Rice has many antioxidant enzymes (such as SOD, CAT, APX, POD, and GR) and antioxidants (such as GSH and AsA) that remove excessive ROS induced by stress [66]. ROS-scavenging metabolic enzyme genes play an important drought resistance role in rice. The overexpression of *CatA*, *CatC*, and *APX* effectively removes ROS in rice and reduces oxidative damage, thus improving drought tolerance [67,68]. The overexpression of *OsAPX2* increases APX activity and enhances the drought tolerance of rice by scavenging ROS [69].

In recent years, a series of functional genes have been identified to improve drought resistance in rice by regulating ROS homeostasis. For instance, IPA1/OsSPL14 positively regulates drought tolerance in rice by directly activating *SNAC1*, which promotes ROS scavenging [70]. The overexpression of *OsSCL30* leads to a significant accumulation of ROS, thus reducing the drought resistance of transgenic rice [71]. OsCBM1 is involved in NADPH oxidase-mediated ROS production by interacting with OsRbohA and OsRacGEF1, thereby enhancing drought tolerance in rice [72]. A mutation of the NADPH oxidase gene *OsRbohB* reduces intracellular ROS production, thus enhancing the sensitivity of rice to drought [73]. The expression levels of *OsSodCc2* and *OscAPX* in *OsDSSR1* overexpression lines increase, and the activities of SOD and APX are enhanced during drought stress, thereby improving drought tolerance [57]. The overexpression of *OsSKIPa* significantly increases the ROS scavenging ability and transcription levels of stress-related genes such as *SNAC1*, thereby enhancing the drought resistance of rice at the seedling and reproductive stages [74]. SOD and POD activities in *OsPUB67* overexpression lines are significantly higher than those in WT, which improves drought resistance by enhancing ROS scavenging ability [75]. The overexpression of *OsLG3* promotes ROS scavenging by regulating downstream antioxidant enzyme-related genes such as *APX1*, *APX2*, *CATB*, *POD1*, *POD2*, and *FeSOD*, thereby significantly improving the drought tolerance of rice [76]. The overexpression of *OsWIH2* inhibits the accumulation of ROS during drought stress, thus significantly improving drought resistance in rice [77]. OsMT1a is directly involved in the ROS scavenging pathway. The CAT, POD, and APX activities of *OsMT1a*-overexpressing transgenic plants are significantly increased, which enhances their drought tolerance [78]. Inhibiting the expression of *OsGRXS17* increases H_2_O_2_ production in guard cells and decreases stomatal aperture, thus improving the drought tolerance of rice [79]. The expression levels of *CatB*, *POD1*, *APX1*, and other antioxidant enzyme genes and the activity of ROS scavenging enzymes in *OsMIOX*-overexpressing transgenic lines are significantly higher than those in WT, which enhances the drought resistance of rice by reducing oxidative damage [80]. OsESG1 is involved in rice drought stress response by regulating antioxidant enzyme activity and stress-regulated gene expression. The inhibition of *OsESG1* expression increases ROS accumulation and decreases the expression and activity of antioxidant enzyme-related genes, such as *OsCAT* and *OsPOD*, under PEG treatment [81]. OsDSM1 is involved in ROS signal transduction. The expression of *POX22.3* and *POX8.1* in the *dsm1* mutant is significantly down-regulated, POD activity decreases, and ROS accumulation increases, thereby increasing the sensitivity of the mutant to drought compared to the WT [82]. The expression of ROS scavenging genes, such as *OsDSM1*, *OsSIK1*, and *OsSKIPa*, and the activity of antioxidant enzymes is significantly enhanced in *OsDIS1*-RNAi transgenic rice. OsDIS1 negatively regulates the drought resistance of rice by inhibiting the antioxidant system to scavenge intracellular ROS [83]. The overexpression of *OsACA6* in transgenic tobacco lines increases ROS scavenging enzyme activity and decreases ROS accumulation, thereby enhancing tolerance to drought stress [84]. Drought stress induces the receptor-like cytoplasmic kinase *OsRLCK241*. The overexpression of *OsRLCK241* enhances ROS detoxification by enhancing the activity of ROS scavengers and the accumulation of compatible osmotic regulators, thereby alleviating osmotic stress caused by drought [85].

### 3.4. Regulation of Hormone Content

Plant growth regulators play an important role in rice adaptation to drought stress. Some functional genes directly regulate the drought stress response of rice by modulating the content and proportion of hormones, such as ABA, ethylene, gibberellin, and cytokinin, in vivo [86].

#### 3.4.1. ABA

ABA is the main drought-responsive hormone. ABA synthesis, metabolism, and signal transduction-related genes regulate drought resistance in rice to varying degrees [87]. Five NCED (9-cis-epoxycarotenoid dioxygenase) genes have been reported in rice [88,89,90]. The overexpression of *OsNCED2* significantly increases the ABA content of transgenic rice at the seedling and reproductive stages, which potentially improves the root development, drought tolerance, and aerobic adaptation of upland rice [91]. The overexpression of *OsNCED3* in *Arabidopsis* increases ABA accumulation and reduces relative water loss, thus enhancing drought resistance [92]. In addition, an ectopic expression of *OsNCED4* in *Arabidopsis* increases ABA content and sugar oversensitivity after seed germination, thus enhancing drought tolerance [93]. OsNCED5 improves the tolerance of rice to water stress by regulating the accumulation of endogenous ABA, which promotes leaf senescence [94]. The overexpression of ABA receptor *OsPYL6* increases ABA accumulation in rice seedlings by up-regulating different NCED genes, which promotes an increase in the total root length and reduced transpiration, leading to enhanced drought tolerance [95]. Similarly, rice cytoplasmic ABA receptor OsPYL5 is a positive regulator of drought stress response, and the constitutive expression of *OsPYL5* improves the drought resistance of rice [96]. RGB1 (rice Gβ subunit) positively regulates ABA biosynthesis by up-regulating the expression of *NCED* genes, which are the positive regulators of ABA response and drought adaptation. The deletion of *RGB1* leads to decreased drought resistance in rice. In contrast, qPE9-1 (rice Gγ subunit) negatively regulates ABA response by inhibiting the expression of vital transcription factors (TFs) involved in an ABA-mediated stress response [97]. The overexpression of a stress-inhibiting gene *OsDSR2* encoding a DUF966 domain-containing protein reduces the sensitivity of rice to ABA-mediated drought resistance by down-regulating the expression of ABA and stress-responsive genes such as *OsNCED4*, *SNAC1*, *Oslea3*, and *rab16C* [98]. *OsABA8ox3* encodes ABA 8′-hydroxylase, which promotes the catabolism of ABA and negatively regulates the drought stress response of rice. The inhibition of *OsABA8ox3* expression thus enhances the drought tolerance of rice [99]. OsASR5 plays multiple roles in rice drought stress responses. It regulates ABA biosynthesis, promotes stomatal closure, and acts as a molecular chaperone to prevent the inactivation of drought stress-related proteins [100]. The overexpression of *OsOLP1* promotes stomatal closure through ABA accumulation, thereby alleviating water loss, which enhances drought tolerance in rice [56]. OsDT11 is involved in an ABA-dependent stress signaling pathway and ABA biosynthesis. *OsDT11*-overexpressing transgenic rice has a significant increase in ABA content and expression of ABA signaling marker genes *BURP*, *GRAM*, and *HVA22*, which positively regulates rice tolerance to drought stress [101].

#### 3.4.2. Other Plant Hormones

The activity of vital enzymes for ethylene synthesis in rice increases significantly during drought, leading to an increase in the ethylene content. This increase consequently promotes organ senescence and branch and leaf abscission, thereby reducing transpiration [102]. Notably, there is a reduction in the expression of vital genes for ethylene synthesis in transgenic rice overexpressing *OsERF109*. OsERF109 modulates the drought resistance of rice by negatively regulating ethylene synthesis [103]. Studies postulate that reducing gibberellin levels can improve plant drought tolerance [104]. The ectopic expression of gibberellin metabolic gene *GA2ox6* (encoding GA2-oxidase) reduces gibberellin content, thus enhancing the drought resistance of rice [105]. Cytokinin (CK) is involved in the regulation of drought resistance in rice. Isopentenyl transferase (IPT) is a vital regulator of CK biosynthesis. The endogenous CK level in transgenic rice overexpressing *IPT* increases, thereby delaying drought-induced leaf senescence [106]. Auxins play an important role in rice responses to drought stress. Transcriptome analysis reveals that the expression levels of numerous auxin-related genes change under dehydration conditions [107]. Overexpression of auxin transport gene *OsPIN3t* and Aux/IAA genes *OsIAA6*, *OsIAA18*, and *OsIAA20* significantly improve the drought resistance of transgenic rice [108,109,110,111]. OsESG1 modulates the initiation and development of rice crown roots by regulating the response and distribution of auxins, thereby inhibiting its expression, which improves the sensitivity of rice to drought stress [81]. Jasmonic acid (JA) and its active derivatives enhance the drought resistance of plants by closing stomata, scavenging ROS, and promoting root development. Drought stress significantly increases the level of endogenous JA [112]. OsJAZ1 negatively regulates the drought resistance of rice via the JA pathway. Of note, the expression levels of many genes in the JA signaling pathway are significantly different between the *OsJAZ1* overexpression lines and WT during drought stress. *OsJAZ1*-overexpressing rice is more sensitive to drought at the seedling and reproductive stage. In the same line, the *jaz1* mutant is more tolerant to drought than the WT [113]. The overexpression of *OsJAZ9* increases the JA level. The JA signal regulates drought stress response via the transcriptional regulation of rice leaf width and stomatal development genes. *OsJAZ9* overexpression lines have narrower leaves, lower stomatal density, and improved drought tolerance [114]. Abscisic acid stress-ripening protein Asr6 regulates the JA-dependent signaling pathway, thus enhancing drought stress in rice. The overexpression of *Asr6* results in the up-regulation of JA biosynthesis-related genes *OsLOX8* and *OsAOS2*, and signal transduction-related gene *COI1*. However, it leads to the down-regulation of *OsJAZ12* [115].

### 3.5. Regulation of Cuticular Wax Deposition

Leaf wax synthesis-related genes modulate the drought resistance of rice by regulating leaf water loss. A mutation of *OsGL1-1* leads to a decrease in cuticle wax deposition, thinning of the cuticle membrane, decrease in chlorophyll leaching, and increase in water loss rate, leading to a decrease in drought tolerance in rice [116]. The overexpression of *OsGL1-2*, a wax synthesis-related gene, increases wax crystals on the leaves of transgenic rice, leading to thickened cuticles and reduced epidermal permeability, thereby significantly enhancing drought resistance [117]. The overexpression of *OsGL1-3* increases wax crystals on the leaf surface, leading to a significant increase in the total load of epidermal wax, which reduces chlorophyll leaching and water loss rates at the seedling and late tillering stage, thereby enhancing the tolerance of rice to water deficit [118]. The inhibition of the expression of *OsGL1-6* significantly reduces wax deposition in the leaf epidermis, causing the cuticle to become thinner, consequently increasing the chlorophyll leaching and water loss rate, which enhances the drought sensitivity of rice [119]. *OsWR1* expression is induced by drought and regulates wax synthesis by changing long-chain fatty acids and alkanes. The overexpression of *OsWR1* increases the expression of genes related to wax/cutin synthesis, thereby reducing water loss and enhancing the drought tolerance of rice [120]. WSL1 prolongs the very long-chain fatty acids (VLCFAs) in rice cuticular wax biosynthesis. The wax crystals on *wsl1*-mutant leaves are sparse, which enhances drought sensitivity [121]. WSL2 is involved in the extension of very long-chain fatty acids. The cuticle of *wsl2*-mutant leaves is thick and less organized, which causes a reduction in the total wax content, thereby enhancing drought sensitivity [122]. WSL5 catalyzes the terminal hydroxylation of alkanes to produce odd primary alcohols involved in the formation of waxy crystals in the epidermis of rice leaves. A *wsl5* mutant has a thicker cuticle and is more tolerant to drought stress [123]. The overexpression of *OsFAR1*, encoding fatty acyl-CoA reductase, reduces leaf permeability and improves the drought resistance of rice by increasing the primary alcohol and total wax content [124]. OsLKP2 interacts with OsGI and negatively regulates wax accumulation on the surface of rice leaves, reducing the recovery ability of rice to drought stress. The cuticular wax content of *oslkp2* or *osgi* mutants increases, thus enhancing their drought tolerance [125]. OsCHR4 regulates cuticular wax formation by epigenetically regulating the expression of wax biosynthesis genes. The expression of seven wax synthesis genes, including *GL1-4*, *WSL4*, and *OsCER7*, in the *oschr4-5* mutant is up-regulated, leading to a significant increase in the wax content of the mutant epidermis, thereby decreasing the water loss rate, which increases drought tolerance [126]. *OsABCG9* mutation significantly reduces the total amount of wax in the leaf epidermis, thus enhancing the drought sensitivity of the mutant [127]. *OsWS1* is regulated by osa-miR1848; the overexpression of *osa-miR1848* down-regulates the *OsWS1* transcript. *OsWS1*-overexpressing plants have increased wax, denser waxy papillae around the stomata, and more cuticle wax crystals on the surface of the leaves and stems, which enhances the drought resistance of rice seedlings [128]. *DWA1* mutation leads to a decrease in the level of ultra long-chain fatty acids. The accumulation of cuticular wax is impaired, and the composition of rice epidermal wax is significantly changed during drought stress, leading to increased drought sensitivity [129].

### 3.6. Regulating Stomatal Density and Stomatal Opening and Closing

The density, size, and opening and closing of leaf stomata are closely associated with the drought resistance of rice [130]. Genes regulating stomatal morphology and opening and closing modulate leaf water loss, thus regulating drought stress response in rice.

Phetluan et al. (2023) used a genome-wide association study (GWAS) to identify nine genes encoding the potential regulatory factors related to stomatal density in 235 rice materials. These genes can be used in rice breeding programs to improve water use efficiency or drought tolerance [131]. The overexpression of rice epidermal model factor *OsEPF1* significantly reduces stomatal density and stomatal conductance, thereby enhancing the drought tolerance of rice [132]. The down-regulation of stomatal development genes *SPCH1*, *MUTE*, and *ICE1* in the dst^Δ184−305^ mutant decreases the stomatal density, which increases leaf water retention, thus improving tolerance to osmotic stress [133]. *EPFL10* mutation reduces stomatal density but does not significantly alter stomatal conductance and carbon assimilation, which leads to higher water preservation than that in wild-type rice and improved drought resilience [134]. *RSD1* mutation results in stomatal cluster distribution, decreased stomatal density, significant down-regulation of stomatal development-related gene *OsSDD1*, and enhanced drought tolerance of the mutant [135].

The overexpression of *OsNHX1* in rice leads to a decrease in the stomatal closure time constant τ_cl_ during drought. The natural variation in the *OsNHX1* gene can be used to regulate the stomatal dynamics of rice, thereby enhancing drought tolerance [136]. The overexpression of *OsASR1* in transgenic rice increases ABA accumulation and regulates stomatal closure and *LEA* gene expression to avoid water loss, thereby enhancing water retention capacity and tolerance to drought stress [137]. OsPUB67 interacts with two negative regulators of drought tolerance, OsRZFP34 and OsDIS1. The overexpression of *OsPUB67* improves drought tolerance in rice by enhancing stomatal closure and ROS scavenging capacity [75]. *DS8* encodes Nck-associated protein 1 (NAP1)-like protein, which plays an important role in the nucleation activity of actin filaments. A damaged actin cytoskeleton in the *ds8* mutant leads to ABA-mediated stomatal closure defects and excessive water loss from leaves, leading to increased drought sensitivity [138]. DCA1 forms a heterotetrameric transcription complex with DST, which negatively regulates stomatal closure by directly regulating the *Prx24* gene associated with H_2_O_2_ homeostasis. The overexpression of *DCA1* thus increases the sensitivity of rice to drought [139].

### 3.7. Improvement in Root Architecture

A deep-penetrating root system and positive geotropic root growth are the main root characteristics of rice to adapt to drought. These two root phenotypes enable rice to absorb water from deeper soil and ensure normal plant growth [140]. *OsSAUR11* encodes a small auxin-up RNA (SAUR) protein, of which the expression is regulated by TF OsbZIP62. OsSAUR11 interacts with the protein phosphatase OsPP36, and its overexpression significantly increases the proportion of deep roots and drought resistance of transgenic rice [141]. *RRS1* encodes an R2R3-type MYB TF that negatively regulates root development by directly inducing *OsIAA3* expression. The silencing of *RRS1* promotes root growth and improves drought resistance in rice [142]. OsFBX257 can bind with kinases OsCDPK1 and OsSAPK2, and its phosphorylation is reversed by the phosphatase OsPP2C08. *OsFBX257* expression regulates the root architecture and drought tolerance of rice. Transgenic lines with inhibited *OsFBX257* expression have a decrease in the total root length, root depth, crown root number, and survival rate during drought stress [143]. OsNMCP1 interacts with the SWI/SNF chromatin remodeling complex to reduce the gene-silencing effect of this complex during drought stress. The overexpression of *OsNMCP1* changes the chromatin accessibility of hundreds of genes, such as *OsNAC10*, *OsERF48*, and *SNAC1*, associated with root growth and drought resistance, resulting in deeper and thicker rice roots and enhanced drought resistance [144]. Ramanathan et al. (2018) postulated that the rice variety Nootripathu had ideal root characteristics to withstand drought and identified the gene *OsARD4*-encoding cis-ketone dioxygenase as the one responsible for the root phenotype. The overexpression of *OsARD4* in the shallow-rooted rice cultivar ASD16 caused the transgenic plants to exhibit similar root growth characteristics to Nootripathu, including faster radicle formation and primary root elongation, earlier crown/lateral root formation, and higher root biomass [145]. The overexpression of *OsERF71* in rice roots alters the root structure, including inducing larger aerenchyma and radial root growth, which improve the drought tolerance of rice [146]. WOX11 interacts with ERF3 to enhance WOX11-mediated crown root growth, which enhances the drought resistance of rice by promoting root hair growth, lateral root initiation, and crown root elongation [147]. Auxins negatively regulate DRO1 and participate in the elongation of root tip cells, resulting in asymmetric root growth and downward root bending in response to gravity. A high expression of *DRO1* increases the growth angle of roots and causes them to grow downward. The introduction of *DRO1* in a shallow root rice variety IR64 caused the transgenic line to withstand drought by increasing the root depth [148]. Reeger et al. (2021) identified *OsLHW*, *AUXIN RESPONSE FACTOR 15*, and *OSH6* as potential TF candidates for regulating the size and number of xylem vessels in rice. Notably, OsLHW is an inhibitor of drought-induced metaxylem plasticity, which can be integrated into a breeding population to improve rice tolerance to drought stress [149].

## 4. Molecular Regulatory Pathways of Genes Associated with Drought Stress Responses in Rice

The molecular regulation of rice response to drought stress has four main levels: transcription, post-transcription, post-translation, and epigenetic modification. Transcriptional regulation occurs mainly through TFs. Post-transcriptional regulation mainly involves microRNAs. Post-translational regulation includes ubiquitination, SUMOylation, phosphorylation, and dephosphorylation processes, while epigenetic regulation involves DNA methylation and histone modification [150]. 

### 4.1. Regulation at the Transcriptional and Post-Transcriptional Levels

#### 4.1.1. Transcriptional Regulation (TFs)

TFs play an important role in stress signal transduction and gene expression regulation [151]. TFs specifically bind to the nucleotide sequence in the promoter region of the downstream gene, thereby regulating its expression. Currently, the TFs involved in drought response in rice include AP2/EREBP, bHLH, bZIP, MYB, NAC, WRKY, and zinc finger proteins (ZFP)(Table 2).

##### AP2/EREBP

The TF encoded by the AP2/EREBP (APETALA2/ethylene-responsive element-binding protein) gene contains a highly conserved AP2/ERF DNA-binding domain, composed of four subfamilies: AP2, DREB (dehydration-responsive element-binding protein), ERF (ethylene-responsive factor), and RAV (related to ABI3/VP1). AP2, DREB, and ERF are subfamilies mainly involved in the drought resistance process of rice.

**Figure 2:**
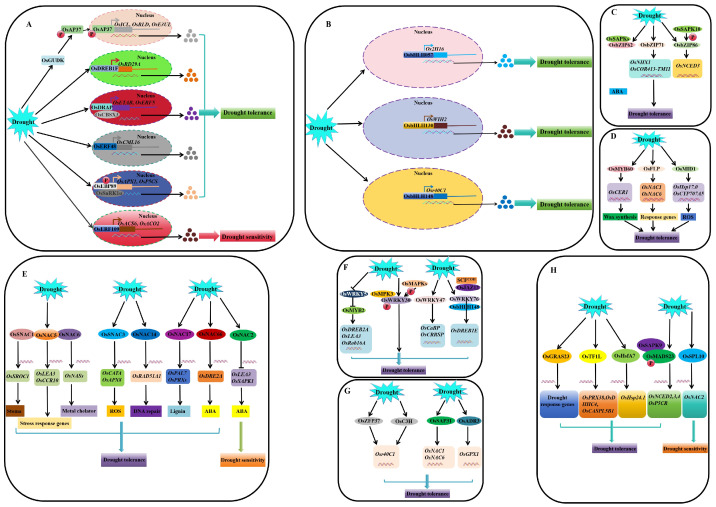
Transcriptional regulation pathway of rice in response to drought stress. (**A**–**G**) OsERFs (**A**), OsbHLHs (**B**), OsbZIPs (**C**), OsMYBs (**D**), OsNACs (**E**), OsWRKYs (**F**), OsZFPs (**G**), and other (**H**) TFs involved in the signal regulation pathway in response to rice drought stress.

Previous studies reported a significant increase in the expression levels of AP2 TF members *OsAP37* and *OsAP59* after 2 h of drought treatment [158,159,160]. Drought-induced receptor-like cytoplasmic kinase GUDK (GROWTH UNDER DROUGHT KINASE) phosphorylates and activates OsAP37, leading to the transcriptional activation of stress-regulation genes, thereby improving the drought tolerance and yield of rice [152,153] (Figure 2A). Notably, the yield of *OsAP59*-overexpressing transgenic rice lines is significantly lower than that of WT under normal and drought conditions, despite the improved drought resistance because of its effect on spikelet development [152].

The OsDREB TF contains an AP2 DNA-binding domain, which specifically binds to DRE/CRT cis-elements to activate the expression of various stress-inducing genes [154]. Water deficit induces the expression of *OsDREB1F*, *OsDREB1G*, *OsDREB2A*, and *OsDREB2B* [155,156]. Transgenic rice overexpressing *OsDREB1A* or *OsDREB1B* has enhanced drought tolerance [154]. OsDREB1F activates the expression of *COR*, *RD29B*, and *RAB18* genes, which contribute to the enhancement of the drought resistance of *OsDREB1F*-overexpressing transgenic rice [155] (Figure 2A). Of note, the overexpression of *OsDREB1G* and *OsDREB2B* significantly increases the tolerance of rice to water deficit, while the overexpression of *OsDREB1E* has no significant effect on the drought tolerance of rice [156]. OsDREB6 has a transcriptional activation activity and specifically binds to the DRE cis-element. *OsDREB6* expression is induced by dehydration, and its overexpression improves the tolerance of transgenic rice to osmotic stress. In contrast, *OsDREB6* RNAi-silencing lines are more sensitive to osmotic stress than WT [157]. *ARAG1*, a DREB gene that responds to ABA levels, is expressed in rice inflorescence, roots, immature embryos, and germinated seeds. The expression level of *ARAG1* increases rapidly under ABA treatment and drought stress. The overexpression of *ARAG1* improves the drought resistance of rice, while *arag1*-knockout mutants are sensitive to drought stress [158]. OsAP21 belongs to the DREB subfamily. Transgenic *Arabidopsis* plants overexpressing *OsAP21* exhibit better growth than WT during drought stress. Moreover, transgenic *Arabidopsis* plants overexpressing *OsAP21* have a significant increase in the proline content, ABA sensitivity, and expression of the early drought response gene *RD29B* [159]. *OsDRAP1* is a DREB2-like gene and is induced by various environmental stresses and plant hormones. The overexpression of *OsDRAP1* during drought stress has a positive effect on maintaining water balance, redox homeostasis, and vascular development of transgenic rice [160]. OsDRAP1 interacts with many genes/proteins and activates numerous downstream drought tolerance-related genes, including important TFs, such as OsCBSX3, in response to drought stress [160].

OsERF71 enhances ABA sensitivity and proline accumulation by promoting the expression of ABA-responsive genes *OsABI5*, *OsPP2C68*, *OsRAB16C*, and *OsRAB16D* and proline biosynthesis genes *OsP5CS1* and *OsP5CS2*, thereby improving drought tolerance in rice [161]. *OsERF101* is induced by drought in leaves, and its overexpression up-regulates ABA-responsive genes *RD22*, *LEA3*, and *PODs*, and increases the proline content and peroxidase activity. *OsERF101* overexpression lines have a higher survival rate and seed-setting rate than WT during the reproductive stage when under drought stress [162]. OsERF109 is localized in the nucleus and possesses transcriptional activation activity. It regulates the expression of ethylene biosynthesis genes *OSACS6*, *OSACO2*, and *OsERF3*, thus negatively modulating the drought resistance of rice [103] (Figure 2A). The ERF TF OsEBP89 interacts with SnRK1α and is phosphorylated. *OsEBP89* silencing induces the expression of stress response-related genes, such as *OsAPX1*, *OsHsfA3*, and *OsP5CS*. It increases the accumulation of proline and ROS scavenging ability, thereby enhancing the tolerance of rice to drought stress during the entire growth stage [163]. The natural variant of the promoter of another ERF family TF, *OsLG3*, is associated with the osmotic stress tolerance of germinated rice seeds. This phenomenon improves the tolerance of rice to simulated drought by inducing ROS scavenging. In addition, the excellent allelic variation of *OsLG3* promotes the improvement in rice drought resistance and is an important genetic resource for breeding drought-tolerant rice varieties [76].

##### bHLH

bHLHs modulate the tolerance or adaptability of plants to environmental stresses by regulating various developmental and metabolic processes of plants, such as photomorphogenesis, flowering induction, and secondary metabolite synthesis [164]. OsbHLH057 positively regulates the drought tolerance of rice by targeting the AATCA cis-acting element in the *Os2H16* promoter [165] (Figure 2B). Drought stress induces the accumulation of OsbHLH130, which consequently activates *OsWIH2* expression. Promoting the biosynthesis of cuticular wax, reducing the water loss rate, and ROS accumulation improves the drought tolerance of rice [77]. *OsbHLH148* is induced by drought stress and acts on the initial response of jasmonates by forming an OsbHLH148-OsJAZ1-OsCOI1 signaling module in the upstream signaling pathway, thereby improving the drought tolerance of rice [166]. In addition, OsbHLH148 modulates the drought tolerance of different rice varieties by regulating *Osr40C1* expression [167] (Figure 2B). The expression of bHLH TF *OsICE1* increases in the roots during drought stress, thereby inducing the expression of the stress response gene *OsWsi18*, which plays an important role in enhancing the drought tolerance of rice at the vegetative and reproductive growth stages [168].

##### bZIP

Genome-wide expression analysis of the rice’s bZIP family reveals that 33 genes (24 up-regulated and 9 down-regulated) are involved in drought response [169,170,171,172]. *OsbZIP10* (OsABF1/OsABI5/OREB1) is involved in drought stress response and ABA signal transduction in rice. Its expression in the shoots and roots of rice seedlings is induced by drought. The up-regulation of ABA/stress regulatory genes, such as *OsNAC*, *OsLEA3*, and *OsABA45*, is significantly inhibited in *osabf1* mutants, causing the mutant to be more sensitive to drought than WT [169]. The expression of *OsbZIP12* is rapidly and strongly induced by drought stress. OsbZIP12 is a positive regulator of ABA signaling and drought tolerance in rice. *OsbZIP12*-overexpressing transgenic rice has increased expression levels of ABA-responsive genes *LEA3* and *Rab16*, resulting in enhanced ABA sensitivity and drought tolerance [170]. *OsbZIP16* is localized in the nucleus and possesses transcriptional activation activity. It is significantly induced by drought stress and positively regulates drought resistance in rice [171]. OsbZIP23 regulates the expression of a series of stress-related genes through an ABA-dependent regulatory pathway. The *osbzip23* mutant has a significant reduction in sensitivity to high concentrations of ABA and tolerance to drought stress [172]. *OsbZIP33* is strongly induced by exogenous ABA and drought stress. The expression levels of downstream drought-inducible genes *LEA7*, *RAB21*, and *RAB16D* in *OsbZIP33*-overexpressing transgenic rice are significantly higher than those in WT during drought. Drought tolerance is enhanced by an ABA-dependent signal transduction pathway [173]. The expression of *OsbZIP42* is induced by ABA, but not by drought. Its activation depends on SAPK4 (stress-/ABA-activated protein kinase 4) and ABA-dependent modification. *LEA3* and *Rab16* are rapidly up-regulated in *OsbZIP42*-overexpressing transgenic rice, and the plants are hypersensitive to ABA and have enhanced drought resistance [174]. OsbZIP46 has high sequence similarity to OsbZIP10 and OsbZIP23, and is strongly induced by drought and ABA treatment. The overexpression of complete *OsbZIP46* has no significant effect on drought resistance because it contains a domain D that has a negative effect on activation. In contrast, the overexpression of *OsbZIP46CA1*, a constitutively active form of OsbZIP46 lacking domain D, activates the expression of downstream stress-related genes and significantly improves the tolerance of rice to drought and osmotic stress [175]. *OsbZIP52* is not induced by drought and ABA. It forms homodimeric complexes, which play a negative regulatory role during drought. *OsbZIP52* overexpression lines demonstrate the down-regulation of abiotic stress-related genes, such as *OsLEA3*, *OsTPP1*, and *Rab25*, which significantly improve their sensitivity to drought stress [176]. *OsbZIP62* expression is induced by drought and ABA treatment. OsbZIP62 binds to the promoters of multiple target genes, interacts with SAPKs, participates in the ABA signaling pathway, and positively regulates the drought tolerance of rice by modulating the expression of stress-related genes [177] (Figure 2C). *OsbZIP66* is up-regulated by an ABA-dependent pathway during drought stress. The overexpression of *OsbZIP66* driven by constitutive and root-specific promoters significantly enhances the drought tolerance of rice [178]. *OsbZIP71* expression is strongly induced by drought and ABA treatment. OsbZIP71 directly binds to the promoters of abiotic stress-related genes *OsNHX1* and *COR413-TM1* in vivo, thus positively regulating the drought tolerance of rice [179]. Transgenic rice overexpressing *OsbZIP72* is hypersensitive to ABA; the expression levels of ABA-responsive genes, such as *LEAs*, are increased, which enhances drought resistance [180]. *OsbZIP86* expression is regulated by miR2105-mediated mRNA splicing. OsbZIP86 binds to the promoter of the ABA biosynthesis gene *OsNCED3*. In the same line, OsSAPK10 phosphorylates and activates OsbZIP86, thus enhancing the expression of *OsNCED3*. The ABA content and drought tolerance of *OsbZIP86*-overexpressing transgenic rice are significantly higher than those of WT during drought [181] (Figure 2C).

Two new bZIP TFs also regulate drought stress responses in rice. *OsHBP1b* transgenic rice exhibits significantly better growth, photosynthetic parameters, and antioxidant enzyme activities than WT during drought conditions. In addition, the root cortex cells of the transgenic lines enlarge and accumulate a large amount of callose, which enhances root penetration into the hard soil and prevents harmful ions from entering the cells [182]. The expression of *EDT1* (ENHANCED DROUGHT TOLERANCE 1), a member of group E of the bZIP TF family in rice, is inhibited by drought stress. Transgenic rice overexpressing *EDT1* has a higher expression level of stress-related genes, such as *OsbZIP12*, *SNAC1*, and *OsLEA3*, which significantly improves their drought tolerance [183].

##### MYB

Although 183 MYB TF family members have been identified in rice, there are limited reports on their functions in drought stress response [184,185,186]. *OsMYB2* expression is induced by drought stress. The overexpression of *OsMYB2* enables rice plants to effectively regulate water potential by accumulating compatible solutes, such as soluble sugar, free proline, and LEA protein, which reduce oxidative damage caused by drought. Moreover, the stress-related genes *OsLEA3*, *OsRab16A*, and *OsDREB2A* are more up-regulated in *OsMYB2*-overexpressing transgenic rice than in the WT, which enhances the drought tolerance of the transgenic rice [184]. The expression of *OsMYB3R-2* is induced by drought stress. Transgenic *Arabidopsis* overexpressing *OsMYB3R-2* has significantly high expression levels of *dehydration-responsive element-binding protein 2A*, *COR15a*, and *RCI2A*, which enhance drought tolerance [185]. *OsMYB6* is induced by drought stress. The overexpression of *OsMYB6* in rice leads to a significantly higher expression of abiotic stress-responsive genes, such as *OsLEA3*, *OsDREB2A*, and *OsDREB1A*, than that in WT, which enhances drought tolerance [186]. *OsMYB48-1* expression is strongly induced by PEG, ABA, and drought. OsMYB48-1 regulates the expression of ABA biosynthesis genes, such as *OsNCED4* and *OsNCED5*, early signaling genes, such as *OsPP2C68* and *OSRK1*, and late responsive genes, such as *RAB21* and *OsLEA3*. The overexpression of *OsMYB48-1* significantly improves the tolerance of rice to mannitol and PEG-simulated drought stress [187]. OsMYB60 directly binds to the promoter region of *OsCER1*, a key regulator of wax biosynthesis. It up-regulates its transcription, promoting the biosynthesis of cuticular wax on the leaf surface, thereby enhancing the drought resistance of rice [188]. OsFLP is an R2R3-MYB transcription activator, which specifically binds to the promoters of *OsNAC1* and *OsNAC6* and positively regulates their expression levels. The overexpression of *OsFLP* significantly improves the drought tolerance of rice [189] (Figure 2C). The *MID1* gene encodes an R-R type MYB TF, which promotes the vegetative growth and reproductive development of drought-stressed rice by up-regulating drought-related genes such as *Hsp17.0*, and *CYP707A5*, and anther development genes, such as *KAR* [190] (Figure 2D).

##### NAC

NAC TFs contain a highly conserved N-terminal DNA-binding domain and a variable C-terminal transcriptional regulatory domain [191,192]. By 2020, more than 170 NAC TFs were reported in rice. The NAC TFs are involved in regulating the drought resistance of rice and have potential application value in breeding new drought-resistant rice varieties [192].

*SNAC1* (*OsNAC9*/*OsNAC19*) is mainly induced in the guard cells by drought. SNAC1 binds to the promoter of *OsSRO1c* to activate its expression, thereby participating in the regulation of stomatal aperture and oxidative stress [191]. Root-specific overexpression of *SNAC1* increases root diameter. These root architecture changes are associated with the up-regulation of the genes involved in ABA and Ca^2+^ signal transduction, lignin and suberin biosynthesis, and cell development and morphogenesis. The root architecture changes enhance the drought resistance of rice during the reproductive growth period, thus promoting an increase in grain yield under drought conditions [191]. SNAC2 (ONAC6/OsNAC6) regulates the expression of target genes *GAT* (involved in membrane modification), *OsNAS1*, *OsNAS2*, and *OsDEP* (involved in nicotinamide (NA) biosynthesis), *GSHT* (involved in glutathione repositioning), *SOT* (involved in the accumulation of 3′-adenosine 5′-phosphate), and *GT* (involved in glycosylation). The drought resistance of rice is enhanced by optimizing root configuration and promoting the accumulation of metal chelating agent NA [192]. However, the growth of transgenic plants with a constitutive overexpression of *SNAC2* is slow, and the yield is reduced [193] (Figure 2D). *SNAC3* (*ONAC003*) expression is induced by drought stress. It regulates the expression of three ROS-related enzyme genes, *CATA*, *APX8*, and *RbohF*, thereby improving the tolerance of rice to drought by regulating ROS homeostasis [194] (Figure 2E).

OsNAC2 regulates ABA and drought stress responses by directly regulating *OsLEA3* and *OsSAPK1*. Of note, the drought resistance of *OsNAC2*-overexpressing rice lines decreases. In contrast, the drought tolerance of RNAi plants improves at the vegetative growth and flowering stages, indicating that OsNAC2 is a negative regulator of drought response [195]. OsNAC5 interacts with OsNAC6 and SNAC1, thereby improving the drought resistance of rice by up-regulating the expression of the drought-induced *OsLEA3* gene without affecting rice growth [192,196,197,198]. OsNAC5 also directly activates *OsCCR10* expression in the roots, promotes lignin accumulation in the thick-walled root tissues and fibers, and improves the drought tolerance of rice by reducing water loss [197] (Figure 2E). In addition, the root-specific overexpression of *OsNAC5* significantly increases the drought tolerance and grain yield of rice at the reproductive growth stage by expanding the roots [198]. Similarly, the root-specific overexpression of *OsNAC10* causes root enlargement, enhanced drought tolerance, and increased grain yield in transgenic plants [199]. OsNAC14 regulates the DNA repair pathway by directly regulating the homologous recombination component *OsRAD51A1*, thereby enhancing the drought tolerance of rice [200]. OsNAC17 is localized in the nucleus, and its expression is significantly induced by drought. It promotes lignin accumulation in the leaves and roots by positively regulating multiple lignin biosynthesis genes, such as *PAL7*, *PRXs*, and *CCR29*, thereby improving the drought tolerance of rice [201]. *OsNAC45*/*ONAC045* is induced by drought and ABA treatment in the leaves and roots. The overexpression of *OsNAC45*/*ONAC045* increases the expression of two stress-responsive genes, *OsLEA3-1* and *OsPM1*, thereby enhancing the drought tolerance of rice [202]. Similarly, *OsNAC58*/*OsNAP* is significantly induced by ABA and drought and mediates rice drought stress response by regulating the expression of ABA-dependent stress response genes [203]. A mutation of *OsNAC092* enhances the ROS scavenging ability of rice under drought stress. The mutant plants also maintain a high GSH/GSSG ratio and redox level to protect the cells from oxidative damage, thereby improving the drought tolerance of rice [204].

ONAC022 is a stress-responsive NAC with transcriptional activation activity. It regulates ABA-mediated drought stress response and drought resistance in rice by regulating ABA biosynthesis genes, such as *OsNCEDs* and *OsPSY*, signal and regulatory genes, such as *OsPP2C02*, *OsbZIP23*, and *OsAP37*, and late stress response genes, such as *OsRAB21*, *OsLEA3*, and *OsP5CS1* [205]. ONAC066 activates the transcription of *OsDREB2A*. The overexpression of *ONAC066* increases rice sensitivity to ABA and tolerance to drought and oxidative stress. However, RNAi-mediated silencing of *ONAC066* leads to contrasting results [206] (Figure 2E). ONAC095 negatively regulates the drought response of rice. The inhibition of *ONAC095* leads to the up-regulation of drought-responsive genes and alters the expression of ABA biosynthesis, metabolism genes, and some ABA signal-related genes, thereby improving the sensitivity of rice to ABA and drought tolerance [207].

##### WRKY

Ramamoorthy et al. (2008) analyzed the rice genome database and predicted 103 genes encoding WRKY TFs. Among them, 19 WRKY genes were regulated by drought [239].

OsWRKY5 directly binds to the promoter region of *OsMYB2* and inhibits its expression, which consequently down-regulates *OsDREB2A*, *OsLEA3*, and *OsRab16A* downstream of OsMYB2 in the ABA signaling pathway. The loss of function of OsWRKY5 increases the sensitivity of rice to ABA, which promotes ABA-dependent stomatal closure, consequently improving the drought tolerance of rice [208] (Figure 2F). The overexpression of *OsWRKY08* in *Arabidopsis* increases the number of lateral roots and the length of the main roots. In the same line, the expression of two ABA-independent abiotic stress response genes, *AtCOR47* and *AtRD21*, in *Arabidopsis* improves the osmotic stress tolerance via the ABA-independent signaling pathway [209]. OsWRKY11 regulates the drought stress response of rice. For instance, the drought tolerance of *HSP101p:OsWRKY11* transgenic lines is significantly improved after heat pretreatment [210]. *OsWRKY30* is induced by drought and ABA and is phosphorylated by interacting with OsMPK3 and other OsMAPKs. The overexpression of *OsWRKY30* improves drought tolerance in rice [211]. OsWRKY45-1 negatively regulates ABA signaling, while OsWRKY45-2 positively regulates ABA signaling. However, the two alleles negatively regulate drought stress response in rice [212]. OsWRKY47 regulates the calmodulin-binding protein gene *CaMBP* and the cysteine-rich secretory protein gene *CRRSP*. The *oswrky47* mutant is sensitive to drought and has reduced yield. In contrast, plants overexpressing *OsWRKY47* have enhanced drought tolerance [213] (Figure 2F). The overexpression of *OsWRKY72* in *Arabidopsis* significantly alters the expression patterns of three auxin-related genes, *AUX1*, *AXR1*, and *BUD1*, in the rosette leaves and inflorescences and two ABA-related genes, *ABA2* and *ABI4*, are expressed, thereby increasing sensitivity to osmotic stress [214]. OsWRKY76 interacts with the OsJAZ protein to activate the transcriptional activity of *OsbHLH148* by interfering with the binding of OsJAZ12 to OsbHLH148. OsWRKY76 and OsbHLH148 directly activate the transcription of *OsDREB1E* during drought stress, thus positively regulating the drought tolerance of rice [215] (Figure 2E). OsWRKY114 negatively regulates the drought stress response of rice by inhibiting stomatal closure. *OsWRKY114*-overexpressing plants significantly down-regulate stomatal closure-related genes *OsPYL2* and *OsPYL10*, which limits stomatal closure, thereby significantly increasing the drought sensitivity of rice [216].

##### Zinc Finger and Zinc Finger-Like TF

Zinc finger and zinc finger-like TF genes associated with response to drought stress in rice include *OsZFP37*, *OsC3H*, *ZFP245*, *ZFP252* (*RZF71*), *DST*, *OsCOIN*, *OsiSAP1*, *OsiSAP7*, *OsiSAP8*, *OsC3H47*, *OsMSR15*, *OsBIRF1*, and *OsRHP1* [167].

The expression of *OsZFP37* and *OsC3H* in the drought-tolerant variety, IR36, is significantly higher than that in the sensitive variety MTU1010. OsZFP37 and OsC3H regulate the drought tolerance of rice varieties via the transcriptional activation of *Osr40C1* [167] (Figure 2G). The ABA signal transduction pathway in rice is activated during drought, causing rapid ZFP245 accumulation. ZFP245 promotes free proline accumulation by inducing the expression of pyrroline-5-carboxylic acid synthase and proline transporter genes, and enhances the ROS scavenging ability of the cells by activating ROS scavenging enzymes [217]. The overexpression of *ZFP252* increases the free proline and soluble sugar content and the expression of stress-defense genes, such as *OsDREB1A*, *OsLEA3*, and *OsP5CS*, thus enhancing the drought resistance of rice [218]. The zinc finger TF DST negatively regulates stomatal closure by directly regulating genes associated with H_2_O_2_ homeostasis. The loss of the *DST* function promotes stomatal closure and reduces stomatal density, thereby enhancing drought tolerance in rice [219]. *OsCOIN* is strongly induced by drought stress, and its overexpression significantly increases the expression of *OsP5CS*, the proline content of cells, and tolerance to drought [220]. OsSAP1 is an A20/AN1 zinc finger protein that interacts with OsAMTR1 and OsSCP [221]. *OsiSAP1*-overexpressing transgenic rice have an altered expression of some genes encoding TFs, membrane transporters, signal components, and genes involved in metabolism, growth, and development, which regulates the tolerance of rice to drought stress at different growth stages [222]. Sharma et al. (2015) investigated the drought stress response of *OsiSAP7* by overexpressing *OsiSAP7* in *Arabidopsis*, driven by a stress-inducible promoter *rd29A*. Transgenic *Arabidopsis* was insensitive to ABA during seed germination but sensitive to drought at the late growth stage, indicating that OsiSAP7 plays a negative regulatory role in ABA and drought stress signals [223]. *OsiSAP8* encodes a cytoplasmic zinc finger protein. *OsiSAP8*-overexpressing transgenic rice has enhanced drought tolerance during the seed germination, seedling, and flowering stages [224]. *OsC3H47* belongs to the CCCH-type zinc finger protein family and can be strongly induced by PEG, ABA, and drought. The overexpression of *OsC3H47* reduces the sensitivity of rice seedlings to ABA but significantly improves the drought resistance of rice [225]. The C2H2-type zinc finger protein OsADR3 enhances antioxidant defense by regulating the expression of *OsGPX1*. It maintains the ASC-GSH cycle by regulating ASC/DHA and GSH/GSSG levels, thereby improving the drought tolerance of rice [226] (Figure 2G). OsMSR15 is also a C2H2-type zinc finger protein that is strongly induced by drought stress in different rice tissues at different developmental stages. The drought tolerance of transgenic *Arabidopsis* is improved by activating the transcription of stress-responsive genes, such as *LEA3*, *RD29A*, *DREB1A*, and *P5CS1* [227]. Unlike most known C2H2-type zinc finger proteins in rice, OsDRZ1 is a transcriptional repressor that enhances the drought tolerance of rice at the seedling stage by maintaining high ROS scavenging enzyme activity and down-regulating drought-responsive genes [228].

*OsBIRF1* encodes a RING-H2 finger protein. Transgenic tobacco with a constitutive expression of *OsBIRF1* exhibits reduced sensitivity to ABA and enhanced drought tolerance during seed germination [229]. *OsRHP1* also encodes a RING-H2 finger protein. The overexpression of *OsRHP1* significantly increases the expression of ABA biosynthesis and ABA-mediated response genes, such as *OsNCED*, *OsABI5*, and *OsLEA1-3*. Increasing the ABA level and enhancing ABA-mediated stress response significantly improves the drought resistance of rice [230].

##### Other TFs Associated with Drought Response

*OsGRAS23* encodes a drought-responsive GRAS TF which binds to the promoters of multiple target genes, regulating the expression of a series of stress-related genes, thereby positively modulating drought tolerance in rice [231]. The rice homeodomain–leucine zipper TF (HD-Zip) OsTF1L directly binds to the promoters of lignin biosynthesis and drought-related genes, such as *poxN*/*PRX38*, *DHHC4*, and *CASPL5B1*, which enhances drought tolerance by increasing lignin accumulation and stomatal closure [232] (Figure 2H). Oshox22 is also an HD-Zip TF, which modulates ABA biosynthesis and negatively regulates drought stress response via the ABA-mediated signal transduction pathway [233]. The heat shock TF (Hsfs) OsHsfA7 is involved in the drought stress adaptation of rice by regulating the target gene *OsHsp24.1* [234] (Figure 2H). OsMADS23 is a stress-responsive MADS-box TF. SAPK9 phosphorylates OsMADS23 by physically interacting with it, thereby improving its stability and transcriptional activity. OsMADS23 promotes the biosynthesis of endogenous ABA and proline by activating the transcription of vital genes, including *OsNCED2*, *OsNCED3*, *OsNCED4*, and *OsP5CR*, which are associated with drought response, thereby positively regulating drought tolerance in rice [235] (Figure 2H). Similarly, OsMADS26 is also a MADS-box TF that acts as an upstream regulator of stress-related genes. The down-regulation of *OsMADS26* enhances rice tolerance to water deficit [236]. OsNF-YA7 is a nuclear factor Y (NF-Y) TF of which the expression is induced by drought stress to regulate drought tolerance in rice in an ABA-independent manner. The 48 genes downstream of OsNF-YA7 action may be involved in the OsNF-YA7-mediated drought tolerance pathway [237]. OsSPL10, a member of the squamosa promoter-binding protein-LIKE (SPL) family, directly regulates the expression of *OsNAC2* as a TF. The inhibition of *OsSPL10* hinders ROS accumulation and programmed cell death, induces rapid stomatal closure, and prevents water loss, thereby improving drought tolerance in rice [238].

#### 4.1.2. Post-Transcriptional Regulation (microRNAs)

MicroRNAs (miRNAs) are small, non-coding regulatory RNAs that regulate various developmental and biochemical processes, including drought stress responses, by promoting the degradation of gene transcripts encoding functional plant proteins [240]. Zhou et al. (2010) identified 30 miRNAs that significantly responded to drought stress in rice. Among the 30 miRNAs, 11 down-regulated miRNAs and 8 up-regulated miRNAs were demonstrated to be induced by drought stress in plants for the first time [241]. Mutum et al. (2016) identified 71 new miRNAs from the drought-tolerant rice variety Nagina 22 [242]. Zhang et al. identified 138 new miRNAs in Dongxiang wild rice (DXWR) (*Oryza rufipogon *Griff.), amongst which 67 were significantly altered under drought stress [243,244]. The targets of drought-responsive miRNAs include TFs, signal receptors, and metabolic enzymes. Drought-responsive miRNAs maintain the growth and development of plants under drought stress by regulating the accumulation of osmolytes, antioxidant defense, hormone metabolism, and other physiological and biochemical processes in rice, thereby improving drought resistance [245].

**Figure 3:**
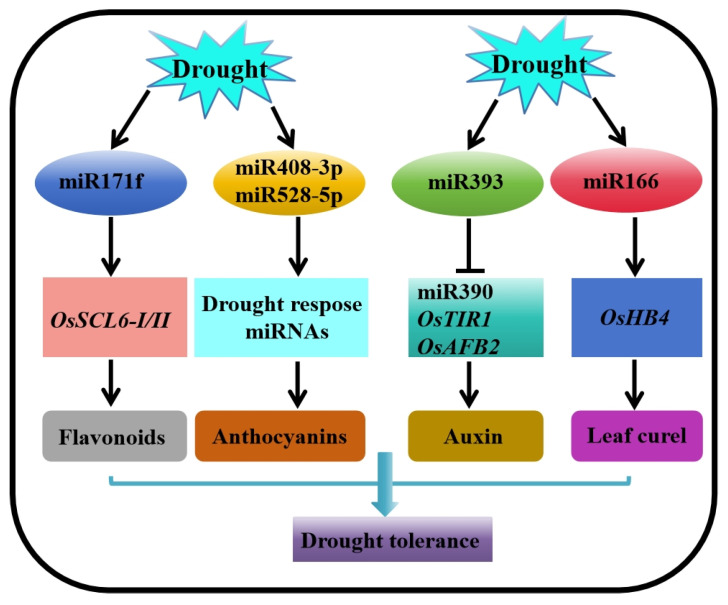
miRNAs involved in the post-transcriptional regulation pathway of rice in response to drought stress.

ABA regulates rice response to drought stress by inducing miR162b and inhibiting its target gene, *OsTRE1*. The knockdown of miR162b or overexpression of *OsTRE1* reduces the accumulation of trehalose and increases the sensitivity of rice to drought [246] (Figure 3). osa-MIR171f regulates the transcription levels of *SCL6-I* and *SCL6-II* and responds to drought by modulating the biosynthesis of flavonoids. Notably, the drought tolerance of osa-MIR171f-overexpressing transgenic plants under field drought and PEG-mediated dehydration stress is higher than that of WT [247] (Figure 3). Drought decreases copper (Cu) levels in the tolerant rice variety Nagina 22. The drought-mediated copper deficiency up-regulates variety-specific drought-responsive miRNAs, such as osa-miR408-3p and osa-miR528-5p, via the TF OsSPL9. This phenomenon leads to the reduction in several transcripts encoding copper-containing proteins, such as anthocyanins, laccases, and Cu/Zn SODs, ultimately promoting ROS accumulation and stomatal closure in tolerant varieties [248]. Osa-MIR2919 regulates the cytokinin and brassinosteroid signaling pathways by modulating 19 target genes, thus negatively regulating drought tolerance [249]. *OsNAC2* is a target of miR164b. The overexpression of miR164b-resistant *OsNAC2* transgenic rice increases the expression levels of ABA biosynthesis and stress-responsive genes, thereby significantly enhancing the drought tolerance of transgenic plants [250]. Under normal growth conditions, auxin-induced miR390 triggers lateral root growth, with miR393 as a potential regulatory factor. Under drought stress, miR393 is induced to regulate miR390-mediated rice lateral root growth negatively [251] (Figure 3). In addition, the overexpression of miR393 inhibits the expression of two rice auxin receptor genes, *OsTIR1* and *OsAFB2*, leading to increased tillering and early flowering but reduced sensitivity to auxin and drought tolerance [252]. Trihelix TFs induce the expression of osa-miR166i-3p during drought stress. osa-miR166i-3p targets HD-ZIP III TF OsHB3, which is involved in leaf senescence, resulting in increased leaf senescence [253]. MiR166 targets OsHB4 TF transcripts. Rice plants overexpressing the miR166-resistant form of *OsHB4* are thus similar to miR166-knockdown lines and exhibit curled leaves and a reduced diameter of the xylem vessels in stems, which significantly improve drought resistance [254] (Figure 3).

### 4.2. Post-Translational Regulation

#### 4.2.1. Ubiquitination and SUMOylation Modification

Plants used ubiquitin and other similar proteins, such as small ubiquitin-related modifiers (SUMOs) to modify target proteins to alter their stability and activity in cells rapidly. The ubiquitin–proteasome system (UPS) regulates multiple signaling pathways by selectively labeling proteins to be degraded by the 26S proteasome [255]. Rice possesses numerous RING (Really Interesting New Gene)-type ubiquitin ligase genes, such as *OsSDIR1* (Salt-And Drought-Induced Ring Finger 1), *OsDIS1* (Drought-Induced SINA Protein 1), *OsRDCP1* (RING domain-containing proteins), and *OsDSG1* (Delayed Seed Germination 1) amongst other genes. These RING-type ubiquitin ligase genes regulate rice resistance to drought stress [256,257,258].

**Figure 4:**
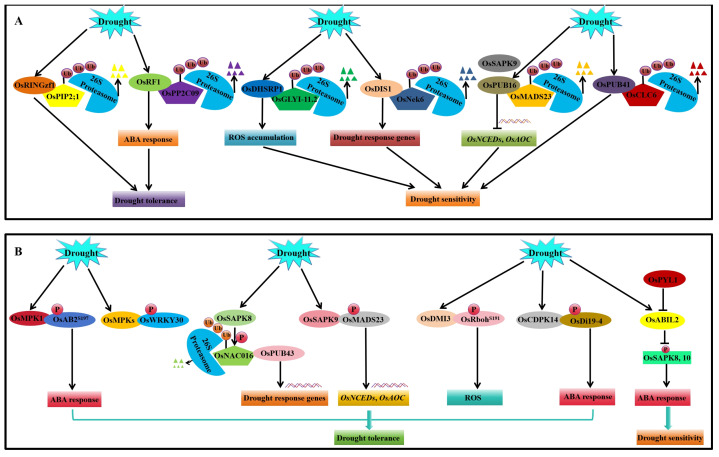
Post-translational regulation pathway of rice in response to drought stress. (**A**) Ubiquitination and SUMOylation regulatory pathways; (**B**) phosphorylation and dephosphorylation regulatory pathways.

OsSDIR1, an E3 ligase-containing ring finger, is expressed in all tissues and is induced by drought stress. *OsSDIR1* transgenic rice exhibits stronger drought tolerance than WT; more stomata are closed, which reduces the leaf water loss rate, thereby increasing the relative water content [258]. *OsRINGzf1* encodes a RING-H2-type E3 ligase, which enhances the water retention capacity of rice through ubiquitination-mediated degradation of the aquaporin OsPIP2-1, thereby positively regulating the drought resistance of rice [37] (Figure 4A). *OsRF1* also encodes RING-H2-type E3 ligase, which positively regulates the ABA signal via the targeted degradation of OsPP2C09. The overexpression of *OsRF1* enhances ABA biosynthesis, promoting endogenous ABA accumulation, thereby improving the drought tolerance of rice [259]. OsDIRP1 and OsDHSRP1 are also E3 ligases-containing ring finger and negatively regulate the drought stress response of rice and *Arabidopsis* [260,261] (Figure 4A). OsDIS1 is a C3HC4 ring finger E3 ligase involved in drought stress signal transduction in rice. OsDIS1 is mainly localized in the nucleus and its expression is induced by drought. The overexpression of *OsDIS1* reduces the drought tolerance of rice, while RNA interference exhibits contrasting results [83]. *OsDIS1*-overexpressing plants regulates numerous drought-responsive genes that interact with tubulin complex-associated serine/threonine protein kinase OsNek6 (NIMA-related kinase 6). OsDIS1 reduces the drought tolerance of rice through the transcriptional and post-translational regulation of various stress-related genes [83]. Drought stress induces the expression of *OsRDCP1*, which enhances the drought tolerance of *OsRDCP1*-overexpressing transgenic rice [257]. The OsDSG1 protein has E3 ubiquitin ligase activity expressed in the leaves and roots and, more significantly, in developing seeds. It is the main regulator of ABA signal during seed germination. *OsDSG1* mutation leads to delayed seed germination, reduced plant height, and enhanced drought resistance [256]. U-BOX protein 16 (OsPUB16) is a U-BOX E3 ubiquitin ligase that negatively regulates drought response in rice. The *ospub16* mutant constructed through CRISPR/Cas9 gene editing produces more endogenous ABA and JA than WT, and its drought tolerance is significantly enhanced [262]. OsPUB16 mediates the ubiquitination degradation of OsMADS23 and inhibits the biosynthesis of ABA and JA by regulating the ‘SAPK9-OsMADS23-OsAOC’ pathway, thereby reducing the drought tolerance of rice [262] (Figure 4A). *OsPUB41* encodes a U-box E3 ligase that is co-localized in the cytoplasm and nucleus of rice cells. Notably, its expression is specifically induced by drought. The chloride channel protein OsCLC6 is a potential substrate of OsPUB41. Rice with silenced or inhibited *OsPUB41* is more tolerant to drought [263]. OsPUB67 is also a U-box E3 ubiquitin ligase that positively regulates the drought resistance of rice [75].

SUMO modifications affect the function, interaction, stability, targeting, and cellular localization of proteins [264]. SUMOylation regulates abiotic stress tolerance such as drought. Three SCE (SUMO-conjugating enzyme) genes in rice are up-regulated during drought. The overexpression of *OsSCE1* affects the biomass and yield, and reduces the drought tolerance of rice [265]. In contrast, *OsSCE3*-overexpressing transgenic rice has enhanced drought tolerance [265]. The constitutive overexpression of rice SUMO E3 ligase gene *OsSIZ1* significantly increases the tolerance of *Arabidopsis* to drought stress [266]. *OsSIZ1* overexpression also enhances the drought and heat tolerance of cotton and significantly improves the yield of cotton under water-saving and rain-fed conditions [267]. Moreover, the heterologous expression of *OsSIZ1* enhances the abiotic stress resistance of creeping bentgrass [268].

#### 4.2.2. Phosphorylation and Dephosphorylation

Phosphorylation is an important post-translational protein modification and an important mechanism of environmental stress signal transduction [269]. Ke et al. (2009) identified 10 drought-related phosphorylated proteins, including NAD-malate dehydrogenase, abscisic acid- and stress-inducible proteins, and ethylene-inducible proteins, amongst other proteins via proteomics [270].

Rice protein kinases, including the MAPK, SnRK2, and CDPK subfamilies, are involved in drought stress response. Currently, 17 MAPKs have been identified in rice. Among them, *OsMPK5*, *OsMPK7*, *OsMPK8*, and *OsMPK12* are drought-inducible genes [271]. Notably, transgenic rice overexpressing *OsMPK5* has been reported to have enhanced drought tolerance [272]. OsMPK1 phosphorylation on the Ser197 site of OsABA2 enhances the stability of the OsABA2 protein, which regulates the biosynthesis of ABA, thereby enhancing the sensitivity of rice to ABA and drought stress tolerance [273] (Figure 4B). OsMPK12/OsBWMK1 is involved in the transduction of plant defense signals through the phosphorylation of OsEREBP1 and OsWRKY33 TFs [274] (Figure 4B). OsWRKY30 interacts with OsMPK3, OsMPK4, OsMPK7, OsMPK14, OsMPK20-4, and OsMPK20-5 and can be phosphorylated by OsMPK3, OsMPK7, and OsMPK14 to activate its transcriptional activity, thereby improving the drought tolerance of rice [211]. OsSAPK3 (Osmotic Stress/ABA-Activated Protein Kinase 3), a member of the SnRK2 family, positively regulates the drought resistance of rice by modulating the accumulation of osmotic adjustment substances, ROS detoxification, and the expression of ABA-dependent and ABA-independent dehydration response genes [275]. During drought stress, ABA accumulation activates SAPK8, which in turn phosphorylates OsNAC016. The phosphorylated OsNAC016 interacts with OsPUB43, resulting in OsNAC016 degradation through the UPS (ubiquitin/26S proteasome system). The degradation weakens the OsNAC016 inhibition of drought-related genes, consequently enhancing the drought tolerance of rice [276] (Figure 4B). SAPK9 is a positive regulator of the ABA-mediated drought stress signaling pathway in rice [277]. SAPK9 phosphorylates OsMADS23 and modulates drought tolerance in rice by regulating ABA biosynthesis [235]. The SAPK9-mediated phosphorylation of OsMADS23 reduces the ubiquitination level of OsMADS23, enhances its stability, and promotes the expression of *OsAOC* by interfering with the OsPUB16-OsMADS23 interaction [262]. Ca^2+^/calmodulin-dependent protein kinase OsDMI3 directly interacts with OsRbohB and phosphorylates the Ser-191 site of OsRbohB. The phosphorylated OsRbohB positively regulates the activity of NADPH oxidase and the production of H_2_O_2_ in ABA signaling, thereby enhancing the sensitivity of seed germination and root growth to ABA and rice tolerance to water stress [262]. *OsCDPK7*, a calcium-dependent protein kinase gene, is induced by drought. *OsCDPK7* overexpression increases the expression of stress-responsive genes, such as *rab16 A*, thus positively regulating the drought tolerance of rice [278]. OsCDPK14 phosphorylates OsDi19-4 and positively regulates ABA response and drought tolerance by regulating the expression of ABA response genes in rice [279]. Leucine-rich repeat receptor-like kinases (LRR-RLKs) and S-like RNases regulate abiotic stress responses [280]. OsPSKR15 is an LRR-RLK which directly interacts with ABA receptors AtPYL9 and OsPYL11 through its kinase domain. An ectopic expression of *OsPSKR15* in *Arabidopsis* increases ABA sensitivity during seed germination, growth, and stomatal closure. A constitutive expression of *OsPSKR15* enhances drought tolerance in *Arabidopsis* by reducing water loss through transpiration [281]. The transcription of LRR-RLK gene *LP2* is directly regulated by the zinc finger TF DST and interacts with drought-responsive aquaporin OsPIP1;1, OsPIP1;3, and OsPIP2;3. *LP2* overexpression reduces the accumulation of H_2_O_2_ and promotes stomatal opening in leaves. Transgenic rice is sensitive to drought stress [282]. The LRR-RLK gene *LRK2* interacts with eukaryotic translation elongation factor 1α (OsEF1A) to regulate cell proliferation, promote branch development, and increase the number of tillers. Transgenic plants overexpressing *LRK2* in the vegetative growth stage have enhanced drought tolerance because of the increase in the number of lateral roots [283]. In addition, LRR-RLK OsASLRK and S-like RNase OsRNS4 synergistically regulate the response of rice to ABA and drought [280]. *OsSIK1* is a putative RLK gene with an extracellular leucine-rich repeat sequence. It positively regulates drought tolerance in rice by activating the antioxidant system [284]. Phosphoenolpyruvate carboxylase kinase (PPCK, EC 4.1.1.32) regulates the activity of phosphoenolpyruvate carboxylase (PEPC, EC 4.1.1.31) by catalyzing the phosphorylation of serine residues at the N-terminus of PEPC, thereby participating in the early response of rice to drought [285,286].

Protein phosphatase-mediated dephosphorylation is an important part of the plant abiotic stress response signaling pathway [287]. The rice PP2C gene *OsPP18* is a downstream target gene regulated by SNAC1. Its expression is induced by drought but has no response to ABA. *ospp18* mutant rice has a higher sensitivity to drought stress at the seedling stage and heading stage than wild-type plants. *OsPP18* overexpression enhances the tolerance of rice to osmotic and oxidative stress [287]. OsABIL2 is a member of clade A PP2C family in rice that negatively regulates ABA signaling and drought resistance in rice. OsABIL2 dephosphorylates SAPK8 and SAPK10. However, the phosphatase activity of OsABIL2 is inhibited by ABA-bound OsPYL1. Phosphorylated SAPK8/10 activates downstream TFs and regulates the expression of ABA-responsive genes, thereby responding to drought stress [288] (Figure 4B). Studies postulate that the OSJNBb0039L24.13 protein, germin-like protein 1 (GLP1), and r40c1 protein are significantly dephosphorylated during drought stress [270]. The OSJNBb0039L24.13 protein plays an important role in signal transduction, while GLP1 is involved in the defense response of some plants. However, GLP1’s specific function in rice has not been reported [289,290].

### 4.3. Epigenetic Regulation

Epigenetic mechanisms, such as DNA methylation and histone modification, play important roles in regulating the expression of drought-responsive genes in rice [291]. DNA methylation is an important epigenetic regulation mechanism that enhances rice adaptation to drought [292]. Single and periodic drought occurrences affect genome-wide DNA methylation [293] and directly or indirectly modulate gene expression through different regulatory pathways [294]. Rice drought stress memory-related differentially methylated regions (DMRs) respond to short-term repeated drought stress by regulating transposon elements and gene expression [292,295]. Notably, there is an up-regulation of four methyltransferase genes in sensitive varieties compared to resistant varieties during drought. In the highlighted study, the promoter and coding region (CDS) methylation levels of CG, CHG, CHH-type CLT1 (chloride transporter), and PSBP (photosystem II polypeptide) were higher, indicating that DNA methylation-driven gene expression confers different drought responses in rice [296]. Gayacharan and Joel (2013) used MSAP (Methylation Sensitive Amplified Polymorphism) technology to quantify cytosine methylation polymorphism in genomic DNA. In the study, drought-sensitive genotypes mainly showed hypermethylation, while drought-tolerant genotypes showed hypomethylation during drought. These findings suggested that hypermethylation is potentially an indicator of drought sensitivity, while hypomethylation is an indicator of drought tolerance. These methylation polymorphisms can be effectively used for rice breeding and the isolation of new drought-responsive genes [292]. In another study, 14 eukaryotic gene superfamily cytochrome P450 genes with different methylation levels were identified within the rice genome of drought-stressed rice [297]. In the same line, a previous study reported demethylation in the region between -1095 and -416 of *C4-PEPC* promoter after 1 h of drought treatment of *C4-PEPC* transgenic rice. The demethylation increased the expression of *C4-PEPC* and the activity of C4-PEPC, thereby promoting drought tolerance in rice [286]. The change in DNA methylation status of genotype-specific genes is associated with the epigenetic regulation of drought stress response [294]. Drought-induced DNA methylation patterns exhibit three characteristics: (1) genotypic specificity, (2) reversibility of most drought-induced methylation/demethylation sites, and (3) significant developmental and tissue specificity. These characteristics play important roles in rice response and adaptation to drought stress [298]. Notably, the DNA methylation pattern of water-saving and drought-resistant rice varieties changes after drought-stress domestication. Differentially methylated loci (DML) are mainly localized in the promoter and exon regions of the gene [299]. The DNA methylation pattern of drought-responsive genes is affected by multi-generational drought and can be inherited between generations. This phenomenon indicates that epigenetic mechanisms play an important role in rice adaptation to upland growth conditions [300].

Histone modification plays a key role in regulating gene expression in plants under abiotic stress [301]. Thousands of genes in rice seedlings undergo different H3K4me3 (Histone H3 lysine4 trimethylation) modifications during drought stress [302]. Zong et al. (2012) used ChIP-Seq and RNA-Seq technology to identify the whole-genome H3K4me3 profiles associated with drought stress in rice [303]. A comparison of the genome-wide differential gene expression pattern with the genome-wide H3K4me3 modification changes revealed a positive correlation between H3K4me3 transcript changes among the genes with both expression and H3K4me3 modification changes in response to drought stress. Of note, the H3K4me3 modification was mainly increased in genes with low expression levels and decreased in genes with high expression levels [303]. Histone H3K4me3 modification and TF OsbZIP23 synergistically regulate drought-responsive genes in rice. H3K4me3 modification upregulates the dehydrin gene and the binding level of OsbZIP23 to the promoter of the dehydrin gene [304]. Transgenic rice overexpressing histone demethylase *OsJMJ703* is sensitive to drought stress. However, RNA interference of *OsJMJ703* enhances the drought resistance of rice [305]. H3K36 methyltransferase SDG708 promotes ABA biosynthesis by directly targeting the vital genes *OsNCED3* and *OsNCED5* involved in ABA synthesis. *SDG708* overexpression enhances drought tolerance and grain yield in rice [306]. HDA704 inhibits the transcription of *DST* and *ABIL2* through histone deacetylation modification. *HDA704* overexpression promotes stomatal closure, reduces the number of stomata, and slows down the rate of water loss, thereby improving the drought tolerance of transgenic rice [307].

## 5. Conclusions and Prospects

Rice requires large amounts of water during planting and is sensitive to drought compared to other crops. Water deficiency affects the growth and development, quality, and yield of rice at multiple levels, including morphological, physiological, biochemical, and molecular changes. Numerous functional genes participate in the drought stress response of rice and the improvement in drought tolerance. These responses and improvements include the promotion of water absorption, increasing the accumulation of osmotic substances, maintaining ROS homeostasis, increasing cuticular wax deposition, reducing stomatal density and opening, and improving root architecture. These functional genes are regulated at the transcriptional, post-transcriptional, and post-translational levels via sophisticated signaling pathways and regulatory networks formed by complex and diverse TFs, miRNAs, and functional enzymes. The biological functions and molecular regulation pathways of drought stress-related genes described in this review provide a theoretical reference for effectively manipulating specific genes and gene clusters during rice breeding programs to enhance drought resistance, thereby boosting rice production and alleviating food insecurity caused by natural and human factors.

This gradual revelation of the molecular mechanism underlying rice drought stress responses forms a theoretical basis for improving rice drought tolerance and breeding new drought-resistant rice varieties. Nonetheless, systematic follow-up studies on various aspects are required to link the theory to the practical. Currently, there are many studies on the phenotypic, physiological, and biochemical aspects of rice roots, stems, leaves, and other organs under drought stress. However, there are only a few specific indicators that can accurately characterize the degree of drought impact based on the different rice varieties, planting environment, and media, which affect the evaluation of rice drought tolerance. For example, the screening of drought-resistant varieties, while factoring in their vigor, plant height, and leaf type under normal conditions, is different. Eliminating background differences in the screening results is thus a challenge that should be solved. In the same line, mining new drought-resistant genes should be carried out to improve the genetic regulatory network of rice in response to drought. Although some drought-related genes have been cloned in rice, the complete regulatory pathways involved in most genes remain unclear. For example, some downstream functional genes, their upstream transcription regulators, and the functional proteins interacting with them are not clear. Numerous TFs have also been reported, but the downstream target genes they directly regulate have not been determined. The incomplete signal transduction pathways limit most reviewed studies, making it impossible to find out the core regulatory factors involved in multiple signal transduction and modifications. There are several ways of cloning and identifying new drought-resistant candidate genes, including (1) using homologous genes of drought-related functional genes reported in model plants (such as *Arabidopsis*) in rice; (2) Another way is through the analysis of omics data, followed by a screening of the TFs or downstream functional genes that significantly respond to drought. The downstream target genes can be preliminarily determined using DAP-Seq or other methods, and the new genes selected and verified using yeast one-hybrid, EMSA, or other biochemical means if the TFs are screened. In the same line, a yeast library of rice drought stress can be constructed, followed by a determination of the upstream TFs using the yeast one-hybrid screening library and verification of the newly selected genes if the functional genes are screened. (3) A rice mutant library can be constructed by screening sensitive and highly resistant mutants to identify new drought-responsive genes through map-based cloning.

Moreover, mining favorable allelic variations in germplasm resources and optimizing the expression patterns of vital genes should be done to provide genetic resources and feasible transgenic strategies for drought-resistant rice breeding. Favorable allelic variations of drought-related genes can be sourced from local germplasm resources or obtained by random point mutation through gene editing. Notably, a constitutive overexpression of some genes with obvious tissue specificities, such as root development and leaf type determination, adversely affects the normal growth of rice. Optimizing the expression patterns of such genes using specific inducible promoters can help achieve better results in establishing their specific functionalities.

## Figures and Tables

**Table 1:** Response of different rice organs to drought.

Location (Organ)	Level	Influence	References
Seed germination (seedlings)	morphological	Buds wither, growth is slowed down, and seed root length and total seedling length are inhibited.	[6,15]
	physiological and biochemical	Water balance is destroyed, membrane transport is damaged, ATP production is reduced, respiration is inhibited, seed germination is delayed and poor. Catalase (CAT), peroxidase (POD), and superoxide dismutase (SOD) activities are changed, and there is an accumulation of free proline.	[6,16,17,18]
Leaves	morphological	Leaf length, width, area, and number, and cell growth and elongation are significantly reduced. The leaves curl, the stomata close, the leaf tip dries or even dies, and the leaf shape changes. There is a reduction in both fresh and dry leaf weight.	[15,19,20,21,22]
	physiological and biochemical	Chlorophyll a, b, a/b, total chlorophyll content, carotenoid content, Fv/Fm, relative water content and membrane stability are significantly reduced, and photosynthesis is inhibited. The leaf water potential is reduced, gas exchange is disturbed, assimilate transport and phloem loading are damaged, relative electrolyte permeability increases, and the distribution of cytokinin and auxin (IAA) changes. There is an accumulation of proline and an increase in CAT, SOD, glutathione reductase (GR), monodehydroascorbate reductase (MDHAR), and dehydroascorbate reductase (DHAR) activity. The content of MDA and H_2_O_2_ increases, and notable changes in nitrogen metabolism indexes, such as nitrogen concentration, glutamine synthetase, and protein content, and carbohydrate metabolism indexes, such as soluble sugar and starch content.	[10,22,23,24,25]
Roots	morphological	There is a decrease in fresh and dry weight, root diameter, xylem vessel diameter or vessel number, and aerenchyma formation. The roots shorten, leading to a decreased biomass. There is an increase in the lateral roots and sclerenchyma cell diameter.	[20,23,26,27]
	physiological and biochemical	Root hydraulic conductivity decreases, xylem sap decreases, and there is a change in root activity, protein, proline, and pigment content. The activities of antioxidant enzymes such as GR, MDHAR, SOD, and DHAR increase.	[22,25,27]
Flowers and grains	morphological	Florets formation is destroyed, resulting in slow grain filling, increased spikelet sterility, and decreased grain weight, size, 1000-grain weight, and seed setting rate. Maturity time is changed, and there is a decrease in biomass and yield.	[19,23,25]
	physiological and biochemical	Starch and amino acid content changes, and there is sugar starvation and an increase in proline content. The activities of ascorbate peroxidase (APX), glutathione (GSH), and ascorbic acid (AsA) increase.	[28,29,30]

**Table 2:** TFs regulating drought stress responses in rice.

Gene Family	Gene	Gene ID	Positive (+)/Negative (−) Regulation	References
AP2/EREBP	*OsAP37*	LOC_Os01g58420	+	[152,153]
	*OsAP59*	LOC_Os02g43790	+	[152]
	*OsDREB1A*	LOC_Os09g35030	+	[154]
	*OsDREB1B*	LOC_Os09g35010	+	[154]
	*OsDREB1F*	LOC_Os01g73770	+	[155]
	*OsDREB1G*	LOC_Os02g45450	+	[156]
	*OsDREB2B*	LOC_Os05g27930	+	[156]
	*OsDREB1E*	LOC_Os04g48350	+	[156]
	*OsDREB6*	LOC_Os09g20350	+	[157]
	*OsARAG1*	LOC_Os02g43970	+	[158]
	*OsAP21*	LOC_Os01g10370	+	[159]
	*OsDRAP1*	LOC_Os08g31580	+	[160]
	*OsERF71*	LOC_Os06g09390	+	[161]
	*OsERF101*	LOC_Os04g32620	+	[162]
	*OsERF109*	LOC_Os09g13940	−	[103]
	*OsEBP89*	LOC_Os03g08460	−	[163]
	*OsLG3*	LOC_Os03g08470	+	[76]
	*OsHYR*	LOC_Os03g02650	+	[164]
bHLH	*OsbHLH057*	LOC_Os07g35870	+	[165]
	*OsbHLH130*	LOC_Os09g31300	+	[77]
	*OsbHLH148*	LOC_Os03g53020	+	[166,167]
	*OsICE1*	LOC_Os11g32100	+	[168]
bZIP	*OsbZIP10*	LOC_Os01g64000	+	[169]
	*OsbZIP12*	LOC_Os01g64730	+	[170]
	*OsbZIP16*	LOC_Os02g09830	+	[171]
	*OsbZIP23*	LOC_Os02g52780	+	[172]
	*OsbZIP33*	LOC_Os03g58250	+	[173]
	*OsbZIP42*	LOC_Os05g41070	+	[174]
	*OsbZIP46*	LOC_Os06g10880	+	[175]
	*OsbZIP52*	LOC_Os06g45140	−	[176]
	*OsbZIP62*	LOC_Os07g48660	+	[177]
	*OsbZIP66*	LOC_Os08g36790	+	[178]
	*OsbZIP71*	LOC_Os09g13570	+	[179]
	*OsbZIP72*	LOC_Os09g28310	+	[180]
	*OsbZIP86*	LOC_Os12g13170	+	[181]
	*OsHBP1b*	LOC_Os01g17260	+	[182]
	*OsEDT1*	LOC_Os05g36160	+	[183]
	*OsMYB2*	LOC_Os03g20090	+	[184]
	*OsMYB3R-2*	LOC_Os01g62410	+	[185]
	*OsMYB6*	LOC_Os04g58020	+	[186]
	*OsMYB48-1*	LOC_Os01g74410	+	[187]
	*OsMYB60*	LOC_Os12g03150	+	[188]
	*OsFLP*	LOC_Os07g43420	+	[189]
	*OsMID1*	LOC_Os05g37060	+	[190]
NAC	*OsSNAC1*	LOC_Os03g60080	+	[191]
	*OsSNAC2*	LOC_Os01g66120	+	[192,193]
	*OsSNAC3*	LOC_Os01g09550	+	[194]
	*OsNAC2*	LOC_Os04g38720	−	[195]
	*OsNAC5*	LOC_Os11g08210	+	[196,197,198]
	*OsNAC6*	LOC_Os01g66120	+	[197]
	*OsNAC10*	LOC_Os11g03300	+	[199]
	*OsNAC14*	LOC_Os01g48446	+	[200]
	*OsNAC17*	LOC_Os03g21030	+	[201]
	*OsNAC45*	LOC_Os11g03370	+	[202]
	*OsNAC58*	LOC_Os03g21060	+	[203]
	*OsNAC092*	LOC_Os06g46270	−	[204]
	*ONAC022*	LOC_Os03g04070	+	[205]
	*ONAC066*	LOC_Os03g56580	+	[206]
	*ONAC095*	LOC_Os06g51070	−	[207]
WRKY	*OsWRKY5*	LOC_Os05g04640	+	[208]
	*OsWRKY8*	LOC_Os05g50610	+	[209]
	*OsWRKY11*	LOC_Os01g43650	+	[210]
	*OsWRKY30*	LOC_Os08g38990	+	[211]
	*OsWRKY45-1*	LOC_Os05g25770	−	[212]
	*OsWRKY45-2*	LOC_Os05g25770	−	[212]
	*OsWRKY47*	LOC_Os07g48260	+	[213]
	*OsWRKY72*	LOC_Os11g29870	−	[214]
	*OsWRKY76*	LOC_Os09g25060	+	[215]
	*OsWRKY114*	LOC_Os12g02400	−	[216]
ZFP	*OsZFP37*	LOC_Os03g38870	+	[77]
	*OsC3H*	LOC_Os09g31482	+	[77]
	*OsZFP245*	LOC_Os07g39870	+	[217]
	*OsZFP252*	LOC_Os12g39400	+	[218]
	*OsDST*	LOC_Os03g57240	+	[219]
	*OsCOIN*	LOC_Os01g01420	+	[220]
	*OsSAP1*	LOC_Os09g31200	+	[221,222]
	*OsiSAP7*	LOC_Os03g57900	−	[223]
	*OsiSAP8*	LOC_Os06g41010	+	[224]
	*OsC3H47*	LOC_Os07g04580	+	[225]
	*OsADR3*	LOC_Os03g55540	+	[226]
	*OsMSR15*	LOC_Os03g41390	+	[227]
	*OsDRZ1*	LOC_Os03g32220	+	[228]
	*OsBIRF1*	LOC_Os02g50930	+	[229]
	*OsRHP1*	LOC_Os08g38460	+	[230]
Others	*OsGRAS23*	LOC_Os04g50060	+	[231]
	*OsTF1L*	LOC_Os08g19590	+	[232]
	*Oshox22*	LOC_Os04g45810	−	[233]
	*OsHsfA7*	LOC_Os01g39020	+	[234]
	*OsMADS23*	LOC_Os08g33488	+	[235]
	*OsMADS26*	LOC_Os08g02070	−	[236]
	*OsNF-YA7*	LOC_Os08g09690	+	[237]
	*OsSPL10*	LOC_Os06g44860	+	[238]

## Data Availability

Not applicable.
